# Association of *SNCA* Parkinson's Disease Risk Polymorphisms With Disease Progression in Newly Diagnosed Patients

**DOI:** 10.3389/fneur.2020.620585

**Published:** 2021-02-10

**Authors:** Aleksandra A. Szwedo, Camilla Christina Pedersen, Anastasia Ushakova, Lars Forsgren, Ole-Bjørn Tysnes, Carl E. Counsell, Guido Alves, Johannes Lange, Angus D. Macleod, Jodi Maple-Grødem

**Affiliations:** ^1^The Norwegian Centre for Movement Disorders, Stavanger University Hospital, Stavanger, Norway; ^2^Department of Chemistry, Bioscience and Environmental Engineering, University of Stavanger, Stavanger, Norway; ^3^Section of Biostatistics, Department of Research, Stavanger University Hospital, Stavanger, Norway; ^4^Department of Clinical Science, Neurosciences, Umeå University, Umeå, Sweden; ^5^Department of Neurology, Haukeland University Hospital, Bergen, Norway; ^6^Department of Clinical Medicine, University of Bergen, Bergen, Norway; ^7^Institute of Applied Health Sciences, University of Aberdeen, Aberdeen, United Kingdom; ^8^Department of Neurology, Stavanger University Hospital, Stavanger, Norway

**Keywords:** *SNCA*, Parkinson's disease, disease progression, genetic association, cognitive impairment

## Abstract

**Objectives:** To evaluate the impact of *SNCA* polymorphisms originally identified as risk factors for Parkinson's disease (PD) on the clinical presentation and progression of the disease in a large cohort of population-based patients with incident PD.

**Methods:** Four hundred thirty-three patients and 417 controls from three longitudinal cohorts were included in the study. Disease progression was recorded annually for up to 9 years using the Unified Parkinson's Disease Rating Scale (UPDRS) or Mini-Mental State Examination. Genotypes for five variants within the *SNCA* locus (rs2870004, rs356182, rs5019538, rs356219, and rs763443) were determined. We studied the association between each variant and disease progression using linear mixed-effects regression models.

**Results:** The clinical profile of the patients with PD at the point of diagnosis was highly uniform between genotype groups. The rs356219-GG genotype was associated with a higher UPDRS II score than A-allele carriers (β = 1.52; 95% confidence interval 0.10–2.95; *p* = 0.036), but no differences were observed in the rate of progression of the UPDRS II scores. rs356219-GG was also associated with a faster annual change in Mini-Mental State Examination score compared with A-carriers (β = 0.03; 95% confidence interval 0.00–0.06; *p* = 0.043).

**Conclusions:** We show that the known PD-risk variant rs356219 has a minor effect on modifying disease progression, whereas no differences were associated with rs2870004, rs356182, rs5019538, and rs763443. These findings suggest that *SNCA* variants associated with PD risk may not be major driving factors to the clinical heterogeneity observed for PD.

## Introduction

Parkinson's disease (PD) is a neurodegenerative disorder characterized by the core motor symptoms, bradykinesia, resting tremor, rigidity, and postural instability, though often accompanied by a wide spectrum of additional motor and non-motor signs (1). The severity and rate of progression of clinical symptoms in PD are highly variable between patients. Some patients experience mild motor decline and non-motor symptoms, whereas some experience fast deterioration in motor symptoms and prominent non-motor symptoms. These differences are in part predicted by sex, age at diagnosis, motor phenotype, and disease severity (2). Similarly, the timing and rate of cognitive decline vary widely among individuals with PD (3), and certain measures, including older age or differences in motor phenotype at diagnosis, predict a more rapid rate of cognitive decline in subgroups of patients (4). The observed heterogeneity can pose prognostic difficulties in a clinical setting, compromising both the planning of appropriate patient management and clinical trial design.

Although heterogeneity in PD is widely recognized, the biological factors modulating the progression remain largely unknown. Association studies have shown that common genetic variance contributes to the risk of developing idiopathic PD (5, 6), and some of these same variants may modify the progression of clinical symptoms (7, 8). The *SNCA* gene encodes α-synuclein, the main protein component of Lewy bodies, which are the pathological hallmark of sporadic PD (9), and genetic variants in the *SNCA* region repeatedly have the strongest association with PD risk in genome-wide association studies (GWASs) (5, 10–12). To date, data on the impact of these *SNCA* polymorphisms on PD progression are scarce, and further investigation in longitudinal studies of patients with PD is needed to refine the link between the genetic variance in *SNCA* and disease course.

Here, we explored the effects of five *SNCA* single nucleotide polymorphisms (SNPs), rs2870004, rs356182, rs5019538, rs356219, and rs763443, on the presentation of PD at the time of diagnosis and the progression of the motor, functional, and cognitive impairment over up to 9 years of regular follow-up, in three deeply phenotyped, longitudinal PD cohorts from Northern Europe.

## Methods

### Study Participants

Three longitudinal cohorts were included in the study: the Norwegian ParkWest study (13), the Parkinsonism Incidence in North-East Scotland (PINE) study (14), and the Swedish New Parkinson Patient in Umeå (NYPUM) study (15). These cohorts provide on-going prospective follow-up of population-based incidence studies of all newly diagnosed PD patients identified in specific geographic regions, initiated between 2002 and 2009. Diagnosis of PD was made according to UK Brain Bank criteria by a neurologist specialized in movement disorders at the baseline visit with continued reassessment at follow-up visits. Participant recruitment and follow-up are summarized in Supplementary Figure 1. Briefly, 605 patients were enrolled: 212 in ParkWest, 211 in PINE, and 182 in NYPUM. Of these, 70 have had a diagnosis other than PD during follow-up, 7 did not consent to follow-up, 57 did not consent to genotyping, and 38 have no DNA sample available, or DNA could not be genotyped. Five hundred twenty-three control subjects were recruited from the same areas: 201 in ParkWest, 266 in PINE, and 56 in the NYPUM study. Of these, 70 have no DNA sample available, or DNA could not be genotyped, 30 did not consent to genotyping, and 6 were diagnosed with PD during follow-up. The remaining 433 PD patients and 417 controls consented to regular follow-up and were eligible for this study. At the time of the study, data from clinical visits for a period of up to 9 years were available (Supplementary Figure 1).

Respective ethical committees approved studies: The Western Norway Regional Committee for Medical and Health Research Ethics, the Multi-Centre Research Ethics Committee for Scotland, and the Regional Ethics Review Board in Umeå. All participants signed written informed consent.

### Clinical Assessment

The clinical assessments have been described in detail, and the same procedures were followed for each cohort (13–15). At baseline, general medical and neurological examinations and semi-structured interviews were performed for all participants to establish medical, drug, and family history (first-degree relative with PD, self-reported). No cases of familial PD were recorded. Patients with PD were assessed at baseline and annual follow-up visits using Hoehn and Yahr staging (16), the Unified Parkinson's Disease Rating Scale (UPDRS) II (activities of daily living) and part III (motor examination) (17), and the Mini-Mental State Examination (MMSE) (18) (in ParkWest, MMSE was evaluated at baseline, the first annual visit and every second year after that), and controls were assessed at baseline and follow-up visits using the MMSE. Home visits were offered to those unable or unwilling to come to the clinic to minimize attrition bias.

Based on subscores of UPDRS III (motor examination), we derived measures of tremor (sum of items 20 and 21), rigidity (sum of item 22), bradykinesia (sum of items 23, 24, 25, 26, and 31), and axial impairment (sum of items 27, 28, 29, and 30). We calculated levodopa-equivalent doses (LEDs) in accordance with published recommendations (19).

### Genotyping of *SNCA* Variants

We selected five *SNCA* polymorphisms (rs2870004, rs356182, rs5019538, rs356219, and rs763443) identified as contributing to a person's risk of developing PD in the largest genome-wide association studies (5, 20) and the largest dedicated genetic study of *SNCA* (6) to date.

Genomic DNA was extracted from peripheral blood using standard methods. Allelic discrimination analysis was performed using predesigned TaqMan SNP genotyping assay (Thermo Fisher Scientific) for rs2870004 (Assay ID: C__26455957_20), rs356182 (C___3208989_10), rs356219 (C___1020193_10), and rs763443 (C___1902284_10) and a custom assay for rs5019538 (Thermo Fisher Scientific). The amplification reactions were performed using the ABI PRISM 7300 Real-Time PCR System (Applied Biosystems) with SDS v1.4 software. The call rates were >99% for each SNP, and the concordance rate was 98%.

### Statistical Methods

All between-group comparisons were performed using IBM SPSS Statistics version 26.0 (Armonk, NY). The regression analysis was done in R version 4.0.2. No differences were observed between the unadjusted and adjusted analyses unless otherwise stated. Two-tailed *p*-values < 0.05 were considered significant, and correction for multiple testing was not performed in this exploratory analysis. As there is insufficient evidence regarding the best genetic model to analyze the effect of *SNCA* SNPs on disease progression, we took an exploratory approach and included both the recessive and dominant genetic models in the analysis plan.

#### Baseline Analysis

Continuous data were summarized using descriptive statistics, whereas categorical data were reported as counts and percentages. Between-group differences in demographic variables were assessed for significance using the Mann–Whitney U tests and χ^2^ tests, as appropriate. Logistic regression (categorical outcome) or linear regression (continuous outcome) was used to test the association between *SNCA* genotypes and PD risk or clinical outcomes at baseline, without and with adjustment for age at baseline and sex. The results of multivariable analyses were presented as odds ratios (ORs) with 95% confidence intervals (CIs) and *p*-values.

#### Longitudinal Analysis

We investigated the association between each of the *SNCA* genotypes and disease progression using three different linear mixed models. The outcome variables for the three models were repeated measurements of UPDRS part II, UPDRS part III, or MMSE total score. MMSE total scores were transformed using log (30 – MMSE + 1) to achieve normality. Time in the study (as a continuous variable) and the *SNCA* genotype (as a binary categorical variable) were included as fixed effects. Patient IDs were included as random intercepts. The interaction between time and the genotype was included as a fixed effect to assess how the *SNCA* genotype influenced disease progression. The analyses were performed without adjustment and with adjustment for the following variables as fixed effects: study cohort, sex, age at baseline, and duration of motor symptoms at baseline. For MMSE, years of education were also included as a fixed effect. For UPDRS II and III, the effect sizes were similar after additional adjustment for LED at each visit (data not shown). Each model had a first-order autoregressive covariance structure. The plot of predictive margins was created using the command margins in Stata 16.00.

## Results

### Baseline Characterization of Study Population

Of the total 850 participants eligible for the study, 433 were patients with PD, and 417 were control subjects (Table 1). The mean age at baseline for PD patients was 69.9 ± 9.6 years, with the proportion of males 60.7%. At the baseline examination, the patients and controls differed with regard to the level of family history of PD (*p* < 0.001), the years of education (*p* = 0.005), and the MMSE score (*p* < 0.001) but not the distribution of sex or age.

**Table 1:** Baseline demographic and clinical characteristics of patients and controls included in study.

**Variable^a^**	**NC**	**PD**	***p* value**
Total, *N*	417	433	
Male, *N* (%)	242 (58.0)	263 (60.7)	0.42
Age at baseline, years	69.6 (±10.2)	69.9 (±9.6)	0.68
Age at first motor symptoms, years		67.9 (±9.6)	
Positive family history, *N* (%)	25 (6.9)	56 (13.0)	**0.005**
Education, years	12.5 (±3.0)	11.2 (±3.6)	**<0.001**
UPDRS II		9.2 (±4.8)	
UPDRS III		24.7 (±11.4)	
Hoehn and Yahr		2.1 (±0.7)	
MMSE, median (IQR)	29.0 (2.0)	29.0 (3.0)	**<0.001**

*NC, normal control; PD, Parkinson's disease; N, count; UPDRS, Unified Parkinson's Disease Rating Scale; MMSE, Mini-Mental State Examination, IQR, interquartile range*.

*Values presented as mean (± standard deviation) unless stated otherwise*.

*Significant p-values (p < 0.05) indicated in bold. P-values calculated using χ^2^ test, Mann–Whitney U-test, as appropriate*.

a *Missing data for family history, 1 PD and 56 NC; education, 20 NC; UPDRS II, 4 PD; MMSE, 76 PD and 7 NC*.

### *SNCA* Variants and Risk of Parkinson's Disease

The distributions of the five *SNCA* SNP genotypes and the minor allele frequencies in PD patients and controls are summarized in Supplementary Table 1. No deviations from Hardy–Weinberg equilibrium were observed for the allele frequencies in patients and controls. Logistic regression analysis was performed to determine if genotype status was associated with a higher incidence of PD, using either the recessive or dominant model (Table 2). In unadjusted analysis, rs356182-G allele carrier status was significantly associated with increased risk of PD compared with noncarriers (OR = 1.33; 95% CI 1.01–1.75; *p* = 0.046). This remained significant after adjustment for age at baseline and sex (OR = 1.32; 95% CI 1.00–1.75; *p* = 0.049). No other significant associations were identified between genotype status and risk of PD.

**Table 2:** Comparison of genotypes of each *SNCA* variant between PD patients and controls.

**SNP**	**Genotype**	**PD, *N* (%)**	**NC, *N* (%)**	**OR**	**(95% CI)**	***p-*value**
**rs2870004**						
Recessive	AA + AT	411 (95.1)	397 (95.7)	1.00		
	TT	21 (4.9)	18 (4.3)	1.13	(0.59–2.15)	0.71
Dominant	AA	275 (63.7)	258 (62.2)	1.00		
	AT + TT	157 (36.3)	157 (37.8)	0.94	(0.71–1.24)	0.66
**rs356182**						
Recessive	AA + AG	363 (83.8)	361 (87.2)	1.00		
	GG	70 (16.2)	53 (12.8)	1.31	(0.89–1.93)	0.17
Dominant	AA	151 (34.9)	172 (41.5)	1.00		
	AG + GG	282 (65.1)	242 (58.5)	1.32	(1.00–1.75)	**0.049**
**rs5019538**						
Recessive	AA + AG	382 (88.4)	371 (89.4)	1.00		
	GG	50 (11.6)	44 (10.6)	1.14	(0.72–1.70)	0.64
Dominant	AA	193 (44.7)	199 (48.0)	1.00		
	AG + GG	239 (55.3)	216 (52.0)	1.14	(0.87–1.50)	0.34
**rs356219**						
Recessive	AA + AG	354 (81.8)	355 (85.3)	1.00		
	GG	79 (18.2)	61 (14.7)	1.30	(0.90–1.87)	0.16
Dominant	AA	141 (32.6)	155 (37.3)	1.00		
	AG + GG	292 (67.4)	261 (62.7)	1.23	(0.92–1.63)	0.16
**rs763443**						
Recessive	CC + CT	331 (76.6)	317 (76.2)	1.00		
	TT	101 (23.4)	99 (23.8)	0.98	(0.71–1.34)	0.89
Dominant	CC	122 (28.2)	105 (25.2)	1.00		
	CT + TT	310 (71.8)	311 (74.8)	0.86	(0.63–1.16)	0.32

*SNP, single nucleotide polymorphism; PD, Parkinson's disease; N, count; NC, normal control; OR, odds ratio; CI, confidence intervals*.

*Significant p-values indicated in bold. P-values calculated using logistic regression with adjustment*.

### *SNCA* Variants and Baseline Parkinson's Disease Profile

Analysis of the association of *SNCA* genotypes and the demographic characteristics of the patients with PD showed no significant differences between groups, except for a higher mean number of years of education for the carriers of rs2870004-TT genotype as compared with rs2870004 A-allele carriers (13.2 ± 4.4 vs. 11.1 ± 3.5 years; *p* = 0.021, Supplementary Table 2). At the time of PD diagnosis, the rs356219-GG genotype was associated with higher UPDRS II scores (*p* = 0.017) (Supplementary Table 2). No differences were shown between baseline clinical presentation of PD and *SNCA* genotypes for the other SNPs investigated (Supplementary Tables 2, 3).

### Effect of *SNCA* Genotypes on Motor and Functional Impairment

**Figure 1:**
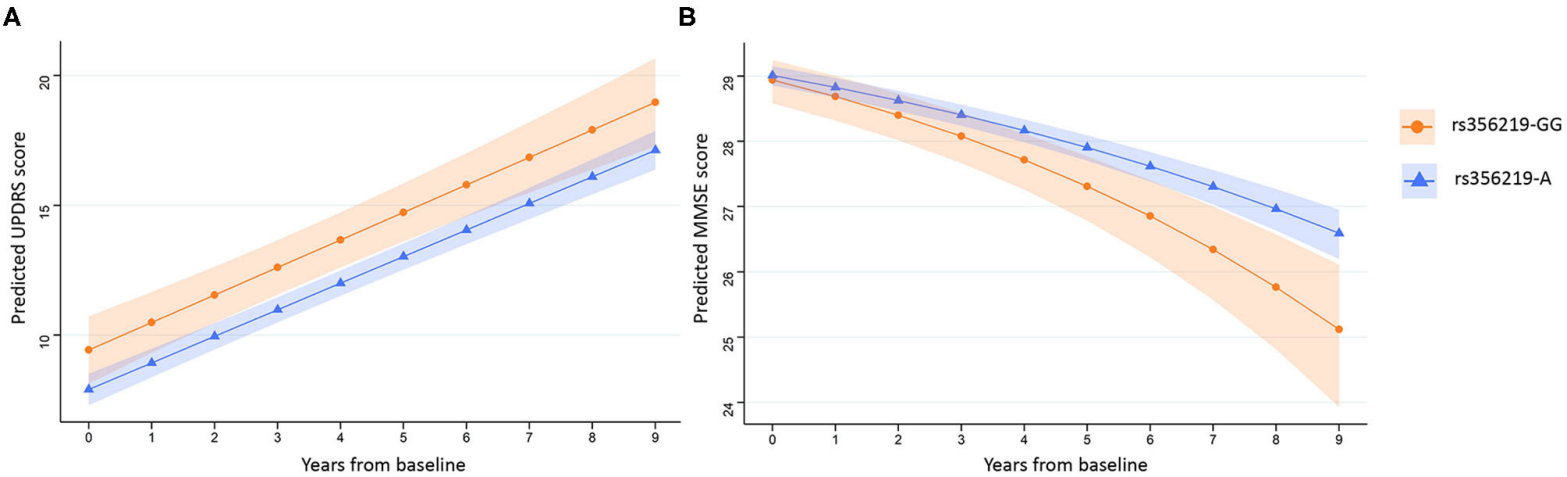
Prediction of UPDRS II and MMSE scores over time. Average predicted UPDRS II **(A)** and MMSE **(B)** scores with confidence bands for first 9 years after diagnosis of PD for rs356219-GG allele carriers (orange, circles) and rs356219-A allele carriers (blue, triangles). UPDRS, Unified Parkinson's Disease Rating Scale; MMSE, Mini-Mental State Examination.

Linear mixed-effects regression analysis with adjustment for age, sex, study cohort, and duration of motor symptoms at baseline revealed that there were no significant differences between any of the *SNCA* genotypes and the rate of annual changes in UPDRS II or III scores measured for up to 9 years (Table 3; Supplementary Table 4). Further adjustment for time-varying LED did not change the significance of the results (data not shown). However, the linear mixed-effects regression analysis reproduced the association between the rs356219 genotype and UPDRS II scores at baseline, with the carriers of rs356219-GG genotype having a 1.52-point higher UPDRS II score during all 9 years of the study in comparison with the carriers of A-allele in adjusted analysis (β = 1.52; 95% CI 0.10–2.95; *p* = 0.036) (Table 3, Figure 1A).

**Table 3:** Association between annual change in clinical assessments of PD and *SNCA* polymorphisms assuming a recessive model.

	**rs2870004^a^**		**rs356182^a^**		**rs5019538^a^**		**rs356219^a^**		**rs763443^a^**	
	**AA + AT vs. TT**		**AA + AG vs. GG**		**AA + AG vs. GG**		**AA + AG vs. GG**		**CC + CT vs. TT**	
	**β (95% CI)**	***p***	**β (95% CI)**	***p***	**β (95% CI)**	***p***	**β (95% CI)**	***p***	**β (95% CI)**	***p***
**UPDRS II^b^**										
Main effect	−0.32 (−2.87; 2.23)	0.81	1.02 (−0.48; 2.52)	0.18	0.82 (−0.91; 2.54)	0.35	1.52 (0.10; 2.95)	**0.036**	−0.50 (−1.80; 0.79)	0.44
Interaction with time	0.13 (−0.30; 0.55)	0.56	0.09 (−0.18; 0.36)	0.52	−0.08 (−0.38; 0.23)	0.63	0.04 (−0.22; 0.29)	0.79	−0.01 (−0.24; 0.21)	0.92
**UPDRS III^b^**										
Main effect	0.27 (−4.82; 5.36)	0.92	0.86 (−2.13; 3.86)	0.57	−0.47 (−3.91; 2.97)	0.79	1.51 (−1.34; 4.36)	0.30	−0.11 (−2.70; 2.47)	0.93
Interaction with time	−0.24 (−1.03; 0.54)	0.55	0.17 (−0.33; 0.66)	0.51	−0.14 (−0.70; 0.41)	0.61	0.08 (−0.39; 0.55)	0.74	−0.29 (−0.70; 0.12)	0.17
**MMSE^c^**										
Main effect	0.03 (−0.29; 0.35)	0.86	0.06 (−0.12; 0.25)	0.50	0.01 (−0.21; 0.22)	0.95	0.03 (−0.14; 0.21)	0.70	−0.12 (−0.27; 0.04)	0.15
Interaction with time	−0.01 (−0.05; 0.04)	0.80	0.02 (−0.01; 0.05)	0.13	0.00 (−0.03; 0.03)	0.90	0.03 (0.00; 0.06)	**0.043**	0.00 (−0.03; 0.02)	0.94

*UPDRS, Unified Parkinson's Disease Rating Scale; MMSE, Mini-Mental State Examination; CI, confidence intervals*.

*Significant p-values (p < 0.05) indicated in bold. Main effect indicates effect of carrier status on intercept, and interaction with time indicates effect of carrier status on slope (change in value per year) of model*.

a *Genotypes grouped according to a recessive genetic model and association with change in clinical assessments assessed using linear mixed models. Reference group is given first*.

b *Adjusted for study cohort, sex, age at baseline, duration of motor symptoms at baseline*.

c *Adjusted for study cohort, sex, age at baseline, duration of motor symptoms at baseline, and years of education at baseline. MMSE score transformed before analysis*.

### rs356219 Is Associated With Faster Cognitive Decline

The rs356219 genotype was associated with a difference in the rate of annual change in MMSE score (Table 3, Figure 1B), with carriers of rs356219-GG predicted to experience a faster decrease in MMSE scores over the 9 years of follow-up compared with an A-allele carrier (β = 0.03; 95% CI 0.00–0.06; *p* = 0.043) after the adjustment for study cohort, sex, age at baseline, duration of motor symptoms at baseline, and years of education. For MMSE, the estimated coefficients cannot be directly interpreted in terms of the annual change in performance, as the data were transformed before analysis. The adjusted model predicts that *SNCA* rs356219-GG carriers would experience on average a fall from 28.9 to 25.1 (95% CI 23.9–26.1) points during 9 years, whereas the MMSE score of A-allele carriers would fall from 29.0 to 26.6 (95% CI 26.2–27.0; *p* = 0.043). Analysis of the association of rs356219 with the rate of change in MMSE score in the control group showed no association between rs356219-GG status and the annual change in MMSE (data not shown). We did not observe any significant effects of the other *SNCA* genotypes on cognitive impairment measured using MMSE (Table 3; Supplementary Table 4).

## Discussion

In this study, we explored the effect of five *SNCA* polymorphisms linked to PD risk on the progression of the disease. Based on the prospective assessment of three population-based incident cohorts of patients with PD, we show an association between rs356219 and the rate of cognitive decline measured from the time of PD diagnosis. The predicted size of the effect of rs356219 on the annual change in cognitive impairment was small, and further, the four other PD risk SNPs investigated had no effect on longitudinal measures of disease severity. Together, these data suggest that although common variants in *SNCA* are important risk factors for PD, these SNPs play a minor role in modifying the progression of PD.

Patients with the rs356219-GG genotype experienced a faster rate of cognitive decline measured by MMSE than A-allele carriers over the 9 years of follow-up. No differences between genotype groups and the annual change in MMSE score were observed for the control subjects over the same follow-up period, indicating that this effect is disease-specific. Similar to our findings, Luo et al. (21) found an association between the rate of cognitive impairment and rs356219. However, in their study of patients with PD from China, carriers of the G-allele had a decreased risk of cognitive decline, indicating that the G allele might have a protective role in this population (21). In an analysis of European patients with PD, Goris et al. reported no association of rs356219 with the annual change in MMSE (22). Notably, this study only followed participants for the first 3.5 years from diagnosis, and based on the predictions from our population, a longer follow-up period would be required to observe the effects of rs356219 on changes in MMSE. In keeping with our findings at the time of PD diagnosis, no difference was observed between rs356219 and mild cognitive impairment in newly diagnosed patients (23). Further, in patients in the later stages of PD (average disease duration at examination 8.8 years), carriers of the rs356219-G allele were at higher risk of cognitive impairment (24). However, a large study analyzing a broad battery of cognitive tests found no association with this SNP in PD patients with a mean of 6.6 years disease duration (25).

Our models predicted that patients with the rs356219-GG genotype would experience on average a one and a half-point larger decrease in MMSE score compared with rs356219-A carriers after 9 years. This small difference suggests that the rs356219 genotype alone is not a strong predictor of cognitive decline in individuals with PD; however, subtle changes of cognitive function may prove to be clinically meaningful in combination with other risk factors. Recently, the rs356219 *SNCA* variant has been suggested to interact in a synergic manner with *GBA* variants to alter the disease course. In a longitudinal study of newly diagnosed patients with PD, rs356219-GG was associated with faster progression to Hoehn and Yahr stage 3 in *GBA*-associated PD but had no detectable effect in noncarriers of a *GBA* variant (26). This indicates that a synergistic interaction between different genetic risk variants could amplify their effect on disease outcomes.

In this study, we did not observe any significant associations between each of the five *SNCA* SNPs and the development of motor or functional impairment. Few studies have analyzed the effect of *SNCA* SNPs on the annual change in UPDRS scores (27, 28), and previously, only the rs356182-GG genotype has been linked to the rate of motor progression, with GG carriers exhibiting a slower rate of change in UPDRS III scores (28). A notable difference to our study is that the patients were not followed from the time of diagnosis but were first examined after a median disease duration of 7 years, and it is possible that the effect of this SNP on modifying motor impairment is more prominent in the later stages of PD. This highlights one of the many difficulties in modeling the relationship between measures of motor impairment and genetic variants, as, in addition to disease duration, the results can also be impacted by differences in the number and frequency of study visits and the length of follow-up. In our study, subjects were followed annually from the time of diagnosis. Although our findings support that common *SNCA* SNPs do not contribute to variability in the rate of motor impairment, it will be important to follow up these findings in the later phases of the disease.

Each of the *SNCA* variants included in our study has been previously linked to the risk of PD (5, 6). In our study population, we observed an association between rs356182 and disease risk under the dominant model. This variant appeared as the top hit with the strongest association with PD risk in consecutive GWASs (5, 10, 11). Two of the SNPs included in our study, rs2870004 and rs5019538, were only recently identified as risk variants for PD in the largest GWASs performed to date (5) or a comprehensive *SNCA* locus study (6) and have not previously been studied in the context of PD phenotype. In this study, we present the first assessment of the disease-modifying effect of these SNPs and find that they do not have a major impact on the presentation or progression of PD. This is in keeping with recent work showing that a genetic risk score based on 31 SNPs associated with the risk of PD was not associated with changes in clinical progression (8).

The present study has notable strengths. All cohorts included in our work are population-based and recruited incident cases representative of the general PD population, as opposed to general research studies, which are generally unrepresentative of the population age distribution of PD (29). Every center used the same standardized diagnostic criteria for PD and clinical outcomes, and patients were all recruited early in the disease and followed prospectively with more than 3,000 study visits. The rate of attrition for reasons other than death was very low, and potential selection bias was minimized by introducing remote visits for those unable to attend clinic visits. The study also has limitations. Firstly, we were only able to include 433 patients with PD and 417 controls, limiting the power of the study to detect small effects, although notably, our study is the largest to date to study the effects of these *SNCA* polymorphisms on disease progression. Furthermore, we acknowledge that our exploratory approach, including five *SNCA* SNPs and two genetic models, increases the risk of false positives. Therefore, the significance of our findings should be interpreted with caution, and this work should be validated. Further, we did not address the potential confounding effect of death on the association with disease outcomes in carriers of these *SNCA* SNPs.

In summary, we report the comprehensive analysis of five *SNCA* PD risk SNPs and their association with long-term disease progression in the largest study to date of patients with PD followed from diagnosis. We find that rs356219 is linked with subtle differences in PD clinical measures, whereas no differences are associated with rs2870004, rs356182, rs5019538, and rs763443, suggesting that these genetic variants do not play a large role in modifying disease progression. This illustrates that PD is a complex disease in which the mechanisms underlying the association of the *SNCA* GWAS signals with PD risk may not be driving factors to the large clinical heterogeneity observed throughout the disease.

## Data Availability Statement

The datasets presented in this study can be found in online repositories. The names of the repository/repositories and accession number(s) can be found below: Novartis supports the publication of scientifically rigorous analysis that is relevant to patient care, regardless of a positive or negative outcome. Qualified external researchers can request access to anonymized patient-level data, respecting patient informed consent, contacting study sponsor authors. The protocol can be accessed through EnCePP portal http://www.encepp.eu/ (EU PAS Register Number EUPAS3247).

## Ethics Statement

The studies involving human participants were reviewed and approved by the relevant ethical committees: The Western Norway Regional Committee for Medical and Health Research Ethics, the Multi-Centre Research Ethics Committee for Scotland and, the Regional Ethics Review Board in Umeå. The patients/participants provided their written informed consent to participate in this study.

## Author Contributions

JM-G, AS, CP, and JL: design of the study. AS, CP, and JM-G: writing the manuscript. LF, CC, GA, and AM: clinical data collection/organization of research project. CP and AS: molecular assays. AS and AU: statistical data analysis. AU, JL, LF, CC, GA, and AM: critical review of the manuscript. All authors contributed to the article and approved the submitted version.

## Conflict of Interest

The authors declare that the research was conducted in the absence of any commercial or financial relationships that could be construed as a potential conflict of interest.
