# Supplementary material for: Association of SNCA Parkinson's Disease Risk Polymorphisms With Disease Progression in Newly Diagnosed Patients
Source: Front Neurol. 2021 Feb 10;11:620585. doi: 10.3389/fneur.2020.620585 (PMC7902914; doi:10.3389/fneur.2020.620585)
Supplement: Supplementary file 1 [file Data_Sheet_1.pdf]

Supplementary materials

Table S1 *SNCA* variants included in the study.

				NC			PD		NC	PD
**SNP**	**Location[a]**	**M/m**	**M/M**	**M/m**	**m/m**	**M/M**	**M/m**	**m/m**	**MAF**	**MAF**
rs2870004	89550094	A/t	258	139	18	275	136	21	0.21	0.21
rs356182	89704960	A/g	172	189	53	151	212	70	0.36	0.41
rs5019538	89715479	A/g	199	172	44	193	189	50	0.31	0.33
rs356219	89716450	A/g	155	200	61	141	213	79	0.39	0.43
rs763443	89898810	C/t	105	212	99	122	209	101	0.49	0.48

SNP, single nucleotide polymorphism; M, major allele; m, minor allele; MAF, minor allele frequency; NC, normal control; PD, Parkinson's disease.

[a] Chromosome 4 location based on SNPdb data base, GRCh38.p12 genome assembly.

Table S2 Baseline characteristics of PD patients included in the study. Genotypes grouped according to a recessive genetic model.

	**rs2870004**		**rs356182**		**rs5019538**		**rs356219**		**rs763443**	
**Variable[a]**	**AA + AT**	**TT**	**AA + AG**	**GG**	**AA + AG**	**GG**	**AA + AG**	**GG**	**CC + CT**	**TT**
Total, N (%)	411 (95.1)	21 (4.9)	363 (83.8)	70 (16.2)	382 (88.4)	50 (11.6)	354 (81.8)	79 (18.2)	331 (76.6)	101 (23.4)
Male, N (%)	246 (59.9)	16 (76.2)	223 (61.4)	40 (57.1)	234 (61.3)	28 (56.0)	215 (60.7)	48 (60.8)	202 (61.0)	60 (59.4)
Age at baseline, years	70.0 (±9.6)	68.5 (±10.3)	69.9 (±9.1)	70.2 (±11.7)	70.0 (±9.5)	69.5 (±10.5)	70.0 (±9.4)	69.7 (±10.6)	69.7 (±9.8)	70.7 (±9.1)
Age at first motor symptoms, years	68.0 (±9.5)	66.2 (±10.2)	67.8 (±9.1)	68.3 (±11.8)	67.9 (±9.5)	67.9 (±10.4)	67.9 (±9.3)	67.7 (±10.6)	68.0 (±9.5)	66.2 (±10.2)
Family history, N (%)	55 (13.4)	1 (4.8)	44 (12.2)	12 (17.1)	48 (12.6)	8 (16.0)	43 (12.2)	13 (10.2)	42 (12.7)	14 (13.9)
Education, years	**11.1 (±3.5)**	**13.2 (±4.4)**	11.2 (±3.5)	11.5 (±3.8)	11.2 (±3.5)	11.5 (±4.0)	11.2 (±3.5)	11.3 (±3.8)	11.2 (±3.5)	11.1 (±3.8)
UPDRS II	9.2 (±4.9)	9.5 (±3.6)	9.1 (±4.7)	10.0 (±5.4)	9.2 (4.9)	9.5 (±4.4)	**9.0 (±4.7)**	**10.4 (±5.4)**	9.3 (±5.0)	8.9 (±4.3)
UPDRS III	24.7 (±11.5)	24.7 (±11.4)	24.6 (±11.3)	25.4 (±12.4)	24.9 (±11.4)	23.4 (±11.7)	24.4 (±11.1)	25.9 (±12.8)	24.7 (±11.8)	24.7 (±10.4)
Hoehn and Yahr	2.1 (±0.7)	2.0 (±0.6)	2.1 (±0.7)	2.1 (±0.8)	2.1 (±0.7)	2.0 (±0.7)	2.1 (±0.7)	2.1 (±0.8)	2.1 (±0.7)	2.1 (±0.7)
MMSE score	28.1 (±2.1)	28.7 (±1.2)	28.2 (±2.1)	28.0 (±2.2)	28.1 (±2.1)	28.2 (±2.0)	28.1 (±2.1)	28.2 (±2.1)	28.1 (±2.1)	28.4 (±1.9)
UPDRS sub-scores										
Tremor	3.4 (±2.9)	3.1 (±3.1)	3.3 (±2.8)	3.7 (±3.2)	3.4 (±2.8)	3.5 (±3.2)	3.3 (±2.8)	3.6 (±3.3)	3.4 (2.9)	3.3 (±2.9)
Rigidity	4.8 (±3.4)	4.9 (±3.5)	4.8 (±3.4)	4.7 (±3.4)	4.9 (±3.4)	4.1 (±3.2)	4.8 (±3.4)	4.8 (3.5)	4.7 (±3.5)	4.9 (±3.1)
Bradykinesia	11.4 (±6.0)	11.3 (±6.4)	11.4 (±5.9)	11.3 (±6.3)	11.4 (±5.9)	11.0 (±6.5)	11.3 (±5.9)	11.7 (±6.5)	11.3 (±6.1)	11.5 (±5.7)
Axial impairment	3.0 (±2.6)	3.1 (±2.1)	2.9 (±2.6)	3.2 (±2.7)	3.0 (±2.6)	2.7 (±2.2)	2.9 (±2.6)	3.2 (±2.7)	3.0 (±2.7)	2.9 (±2.3)

N, count; UPDRS, Unified Parkinson's Disease Rating Scale; MMSE, Mini Mental State Examination.

Values presented as mean (± standard deviation) unless stated otherwise.

Groups comparisons with significant *p*-values ($p < 0.05$) indicated in bold. P-values calculated using χ^2 tests, Mann-Whitney U or linear regression, as outlined in the methods.

[a] Missing data for: family history, 1; UPDRS II, 4; MMSE, 76.

Table S3 Baseline characteristics of PD patients included in the study. Genotypes grouped according to a dominant genetic model.

	**rs2870004**		**rs356182**		**rs5019538**		**rs356219**		**rs763443**	
**Variable [a]**	**AA**	**AT + TT**	**AA**	**AG + GG**	**AA**	**AG + GG**	**AA**	**AG + GG**	**CC**	**CT + TT**
Total, N (%)	275 (63.7)	157 (36.6)	151 (34.9)	282 (65.1)	193 (44.7)	239 (55.3)	141 (32.6)	292 (67.4)	122 (28.2)	310 (71.8)
Male, N (%)	164 (59.6)	98 (62.4)	82 (54.3)	181 (64.2)	111 (57.5)	151 (63.2)	78 (55.3)	185 (63.4)	73 (59.8)	189 (61.0)
Age at baseline, years	70.1 (±9.8)	69.7 (±9.2)	69.5 (±9.6)	70.2 (±9.6)	69.6 (±9.1)	70.2 (±10.0)	69.4 (±9.2)	70.2 (9.8)	69.0 (±10.3)	70.3 (±9.3)
Age at first motor symptoms, years	68.0 (±9.8)	67.7 (±9.2)	67.5 (±9.6)	68.1 (±9.6)	67.6 (±9.1)	68.1 (±10.0)	67.4 (±9.2)	68.1 (±9.8)	67.0 (±10.3)	68.3 (±9.3)
Positive family history, N (%)	37 (13.5)	19 (12.2)	14 (9.3)	42 (14.9)	23 (12.0)	33 (13.8)	15 (10.7)	41 (14.0)	14 (11.5)	42 (13.6)
Education, years	11.2 (±3.5)	11.2 (±3.6)	11.3 (±3.4)	11.2 (±3.6)	11.6 (±3.7)	10.9 (±3.4)	11.6 (±3.6)	11.1 (±3.6)	11.6 (±3.8)	11.1 (±3.4)
UPDRS II	9.4 (±4.9)	8.9 (±4.7)	9.1 (±4.9)	9.3 (±4.8)	9.2 (±4.8)	9.2 (±4.9)	9.1 (±4.8)	9.3 (±4.8)	9.7 (±4.4)	9.0 (±5.0)
UPDRS III	25.1 (±11.6)	24.0 (±11.1)	23.9 (±11.8)	25.1 (±11.2)	23.8 (±10.9)	25.4 (±11.8)	24.2 (±11.5)	24.9 (±11.4)	24.9 (±11.4)	24.6 (±11.5)
Hoehn and Yahr	2.1 (±0.7)	2.1 (±0.7)	2.1 (±0.7)	2.1 (±0.7)	2.1 (±0.7)	2.1 (±0.7)	2.1 (±0.7)	2.1 (±0.7)	2.1 (±0.7)	2.1 (±0.7)
MMSE score	28.1 (±2.0)	28.1 (±2.1)	28.3 (±1.8)	28.1 (±2.2)	28.2 (±2.0)	28.1 (±2.2)	28.4 (±1.7)	28.0 (±2.2)	28.1 (±1.9)	28.2 (±2.1)
UPDRS sub-scores										
Tremor	3.5 (±2.8)	3.3 (±3.0)	3.3 (±2.6)	3.4 (±3.0)	3.2 (±2.7)	3.5 (±3.0)	3.2 (±2.5)	3.5 (±3.0)	3.4 (±2.9)	3.4 (±2.9)
Rigidity	4.8 (±3.4)	4.7 (±3.3)	4.7 (±3.4)	4.8 (±3.4)	4.6 (±3.3)	4.9 (±3.5)	4.7 (±3.5)	4.8 (±3.3)	4.8 (±3.4)	4.8 (±3.4)
Bradykinesia	11.6 (±6.1)	10.9 (±5.9)	11.1 (±6.1)	11.5 (±5.9)	11.0 (±5.7)	11.6 (±6.2)	11.4 (±5.9)	11.4 (±6.1)	11.3 (±6.0)	11.4 (±6.0)
Axial impairment	3.0 (±2.6)	2.8 (±2.5)	2.8 (±2.6)	3.0 (±2.5)	2.8 (±2.7)	3.1 (±2.5)	2.8 (±2.6)	3.0 (±2.6)	3.0 (±2.8)	2.9 (±2.5)

N, count; UPDRS, Unified Parkinson's Disease Rating Scale; MMSE, Mini Mental State Examination.

Values presented as mean (± standard deviation) unless stated otherwise.

Groups comparisons with significant *p*-values ($p < 0.05$) indicated in bold. P-values calculated using χ^2 tests, Mann-Whitney U, or linear regression, as outlined in the methods.

[a] Missing data for: family history, 1; UPDRS II, 4; MMSE, 76.

Table S4 The association between annual change in clinical assessments of PD and *SNCA* polymorphisms assuming a dominant model.

	**rs2870004** [a] **AA** versus AT + TT		**rs356182** [a] **AA** versus AG + GG		**rs5019538** [a] **AA** versus AG + GG		**rs356219** [a] **AA** versus AG + GG		**rs763443** [a] **CC** versus CT + TT	
	**β (95% CI)**	***p***	**β (95% CI)**	***p***	**β (95% CI)**	***p***	**β (95% CI)**	***p***	**β (95% CI)**	***p***
**UPDRS II** [b]										
Main effect	0.64 (-0.50; 1.78)	0.27	0.13 (-1.02; 1.28)	0.83	0.01 (-1.09; 1.11)	0.96	0.00 (-1.17; 1.17)	1.00	0.94 (-0.28; 2.15)	0.13
Interaction with time	-0.12 (-0.32; 0.07)	0.22	-0.10 (-0.30; 0.10)	0.32	-0.09 (-0.28; 0.10)	0.36	-0.11 (-0.31; 0.09)	0.27	-0.16 (-0.37; 0.05)	0.14
**UPDRS III** [b]										
Main effect	1.03 (-1.25; 3.30)	0.38	-0.20 (-2.49; 2.10)	0.87	-1.20 (-3.40; 1.00)	0.29	0.04 (-2.30; 2.38)	0.97	0.84 (-1.59; 3.27)	0.50
Interaction with time	-0.14 (-0.50; 0.22)	0.44	0.10 (-0.27; 0.47)	0.60	0.09 (-0.26; 0.44)	0.62	-0.07 (-0.44; 0.30)	0.71	-0.01 (-0.39; 0.38)	0.98
**MMSE** [c]										
Main effect	0.00 (-0.14; 0.13)	0.95	-0.06 (-0.20; 0.08)	0.37	-0.01 (-0.14; 0.12)	0.89	-0.07 (-0.21; 0.07)	0.34	0.06 (-0.08; 0.21)	0.40
Interaction with time	0.00 (-0.02; 0.02)	0.82	0.01 (-0.02; 0.03)	0.60	0.00 (-0.02; 0.02)	0.90	0.01 (-0.02; 0.03)	0.57	0.00 (-0.02; 0.02)	0.90

UPDRS, Unified Parkinson's Disease Rating Scale Part; MMSE, Mini Mental State Examination; CI, confidence intervals.

The main effect indicates the effect of carrier status on the intercept and the interaction with time indicates the effect of carrier status on the slope (change in value per year) of the model.

[a] Genotypes grouped according to a dominant genetic model and the association with change in clinical assessments assessed using linear mixed models. The reference group is indicated in bold.

[b] Adjusted for study cohort, sex, age at baseline, duration of motor symptoms at baseline.

[c] Adjusted for study cohort, sex, age at baseline, duration of motor symptoms at baseline and years of education at baseline. MMSE score transformed before analysis.
